# Resazurin-based 96-well plate microdilution method for the determination of minimum inhibitory concentration of biosurfactants

**DOI:** 10.1007/s10529-016-2079-2

**Published:** 2016-03-11

**Authors:** Mohamed Elshikh, Syed Ahmed, Scott Funston, Paul Dunlop, Mark McGaw, Roger Marchant, Ibrahim M. Banat

**Affiliations:** School of Biomedical Sciences, Pharmaceutical Science and Practice Research Group, Ulster University, Cromore Road, Coleraine, County Londonderry BT52 1SA Northern Ireland, UK

**Keywords:** Antibiotics, Biosurfactant, Minimum inhibitory concentration, Resazurin

## Abstract

**Objectives:**

To develop and validate a microdilution method for measuring the minimum inhibitory concentration (MIC) of biosurfactants.

**Results:**

A standardized microdilution method including resazurin dye has been developed for measuring the MIC of biosurfactants and its validity was established through the replication of tetracycline and gentamicin MIC determination with standard bacterial strains.

**Conclusion:**

This new method allows the generation of accurate MIC measurements, whilst overcoming critical issues related to colour and solubility which may interfere with growth measurements for many types of biosurfactant extracts.

## Introduction

Research into the biomedical properties of biosurfactants has shown that these compounds demonstrate potency as antimicrobial agents (Rodrigues et al. 2006). The minimal inhibitory concentration (MIC), which is a key indicator of an antimicrobial agent’s potency, is defined as the concentration (mg l^−1^) at which visible growth of bacteria is prevented under defined growth conditions (Wiegand et al. 2008). Well- and disc-diffusion methods have frequently been reported as qualitative indicators for testing the antimicrobial activity of natural products (Yemoa et al. 2011). Such testing methods are standardised by the Clinical and Laboratory Standards Institute (CLSI) for antibiotic testing (The Clinical and Laboratory Standards Institute M100-S22, Volume 32 No 3. January 2012).

Currently, there are no interpretational criteria for test results of natural products when using the disc diffusion method and these can only offer zone of inhibition indicative outcomes. Furthermore, some technical problems may contribute to the lack of accuracy of these methods, such as the polarity of the natural compound which may play a role in the extent of diffusion (Sanchez and Kouznetsov 2010). Other issues may reduce the efficiency of these methods such as precipitation out of solution of the biosurfactants in the case of lactonic sophorolipids (Weber et al. 2012), and also our own observation of the lack of solubility of rhamolipid C_14_-C_14_ produced from *Burkholderia thailandensis* E264.

**Fig. 1:**
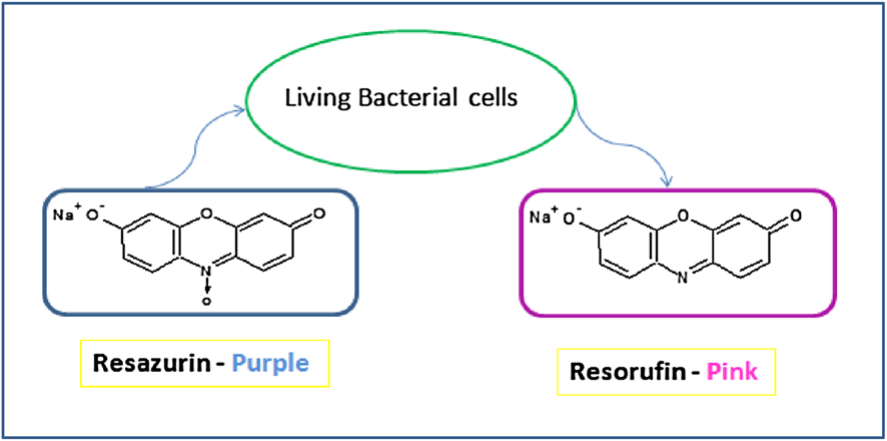
Active living cells cause reduction of resazurin (*purple-blue*) to resorufin (*pink-colorless*)

Microdilution and agar dilution are quantitative methods that can be used to determine MIC values (Kim et al. 2007). Although results obtained with the agar dilution method show good correlation with the microdilution method (Amsler et al. 2010), the agar dilution method is laborious and time consuming and more importantly the factors that adversely affect the disc diffusion method may also contribute to the lack of accuracy with the agar dilution method especially when dealing with sparingly soluble biosurfactants. On the other hand, the microdilution method is standardised, accurate, inexpensive to perform, and easy to carry out (Jorgensen and Ferraro 2009). The improved microdilution method described in this report is enhanced through the addition of resazurin dye as a redox indicator, which overcomes the problems associated with sparingly soluble test materials. Active bacterial cells reduce the non-fluorescent resazurin (blue) to the fluorescent resorufin (pink) which can be further reduced to hydroresorufin (O’Brien et al. 2000) as shown in Fig. 1, giving a direct quantifiable measure of bacterial metabolic activity.

## Materials and methods

### Method validation

To establish the accuracy of this method, it was first important to validate the performance of standard antibiotics against several ATCC strains to determine the MIC values and compare them with those published by The Clinical and Laboratory Standards Institute (M100-S22, Volume 32 No 3. January 2012). In this assay, two of the quality control ATCC strains, namely Gram-positive *Staphylococcus aureus* ATCC 29213 and Gram-negative *Escherichia coli* ATCC 25922, were screened against the antibiotics tetracycline (bacteriostatic in action) and gentamicin (bactericidal in action) and the MIC determined through recording of the colour change observed.

### Testing reagents

Tetracycline hydrochloride (Sigma), gentamicin sulphate (Sigma), Muller-Hinton broth (Oxoid), Muller Hinton agar (Oxoid), Resazurin (Sigma), rhamnolipids JBR325 comprising approx. 3:1 dirhamnolipids to monorhamnolipids respectively (Jeneil Biotech), Rhamnolipids (produced in-house from *Burkholderia thailandensis* E264 predominantly comprising C_14_–C_14_ dirhamnolipid with a minor component of monorhamnolipid), lactonic sophorolipids (LSL) mainly comprising the diacetylated congener of molecular weight 688, acidic sophorolipids (ASL) comprising different chain length acetylated congeners, Polymyxin B sulphate comprising a mixture of poymyxin B1 and B2 (Sigma).

### Test microorganisms

*Streptococcus mutans* (DSM-20523)*; Streptococcus oralis* (DSM-20627); *Actinomyces naeslundii* (DSM-43013)*; Neisseria mucosa* (DSM-4631)*; Methicillin resistant Staphylococcus aureus* (*MRSA*) (ATCC14576) and *Streptococcus sanguis* (NCTC 7863) were used.

### Preparation, storage and testing reagents

Resazurin was prepared at 0.015 % by dissolving 0.015 g, vortexed and filter sterilised (0.22 µm filter) and stored at 4 °C for a maximum of 2 weeks after preparation.

### Preparation of standardised inoculum

The inocula were prepared in accordance with the CSLI recommendation where the OD_600_ value was adjusted to the equivalent of 10^8^ CFU ml^−1^, which was determined from a calibration curve for each microorganism.

### Preparation of 96 well-plates for testing biosurfactants or standard antibiotics

**Fig. 2:**
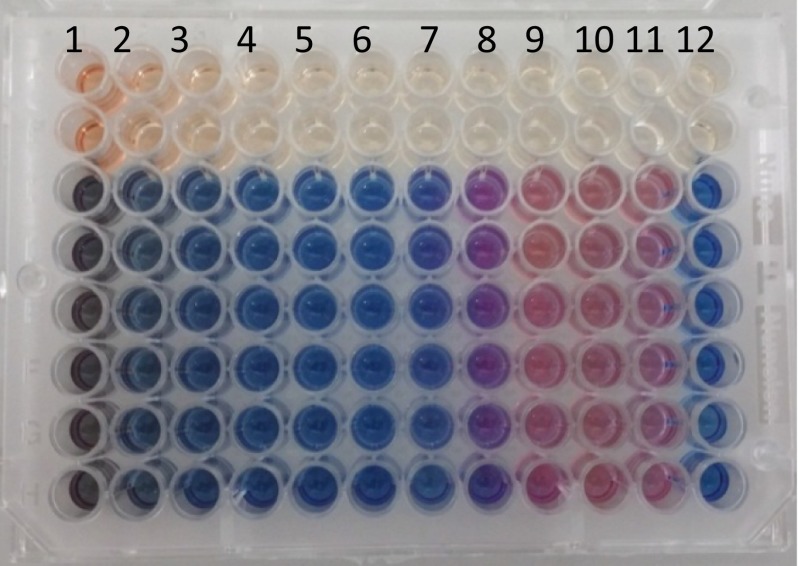
Determination of MIC for Rhamnolipid JBR325 against *Streptococcus mutans* (DSM-20523). After the period of incubation, resazurin dye was added. Column 12 confirms no contamination occurred while preparing the plate. Column 11, a negative control shows a change of resazurin natural colour (*blue/purple*) to the reduced form (*red-colourless*). The highest concentration incorporated into the plate is 25 mg ml^−1^ and the lowest achieved through double serial dilution is 0.05 mg ml^−1^. Column 7 shows no colour changes therefore concentration of biosurfactant in that column was taken as the MIC value. The range of biosurfactant concentration in the wells was 25–0.05 mg ml^−1^

Biosurfactants were dissolved in Muller Hinton broth (MHB) at twice the concentration of the final test, with pH adjusted to 7. 100 µl of the biosurfactant/MHB broth was dispensed in each well of Column 1, while Columns 2-10 contained 50 µl of MHB broth only. Column 11 contained 100 µl of diluted standardised inoculum, and Column 12 contained 100 µl of the medium broth (as a control to monitor sterility), as shown in processed plate Fig. 2. A multichannel pipette was then used to transfer and mix biosurfactants from column 1–10, resulting in 50 µl biosurfactant per well. The tested concentrations of the different biosursurfants achieved through double serial dilutions from columns 10–1 were as follows; 25–0.05 mg ml^−1^ rhamnolipids (JBR325), 50–0.01 mg ml^−1^ rhamnolipids from *Burkholderia thailandensis* E264, 12.5–0.025 mg ml^−1^ of lactonic sophorolipids, 100–0.02 mg ml^−1^ of acidic sophorolipids and 1–0.002 µg ml^−1^ of polymyxin. The standardised microorganism suspension was then diluted by 1:100 in MHB broth. 50 µl of the adjusted OD_600_ bacterial suspension was then added to all wells containing biosurfactant and to the control wells, resulting in approx. 5 x10^5^ CFU ml^−1^. The time taken to prepare and dispense the OD adjusted bacteria did not exceed 15 min. After incubation for 24 h at 37 °C, resazurin (0.015 %) was added to all wells (30 µl per well), and further incubated for 2–4 h for the observation of colour change. On completion of the incubation, columns with no colour change (blue resazurin colour remained unchanged) were scored as above the MIC value. The minimum biocidal concentration (MBC) was determined by plating directly the content of wells with concentrations higher than the MIC value, as detailed in Table 1. The MBC value was determined when there was no colony growth from the directly plated contents of the wells. In addition the contents of the wells showing indications of growth inhibition were serially diluted to quantify an end-point killing of the bacteria as detailed in the results section.

**Table 1:** Determination of the MIC by Resazurin aided microdilution method of two antibiotics against two standard strains

Bacteria	Antibiotic	MIC reported in this study (µg ml^−1^)	MIC recommended by CLSI (µg ml^−1^)
*Escherichia coli* ATCC 25922	Tetracycline	2	0.5–2
*Staphylococcus aureus* ATCC 29213	Tetracycline	0.5	0.12–1
*Escherichia coli* ATCC 25922	Gentamicin	1	0.25–1
*Staphylococcus aureus* ATCC 29213	Gentamicin	0.5	0.12–1

Values obtained were compared with those recommended by the CLSI

## Results and discussion

Results obtained from the validation assay were in close agreement with the recommendations of CLSI as shown in Table 1. Different biosurfactants were tested to determine their MIC and MBC values as shown in Table 2. It is clear that the biosurfactants varied in their efficacy from no effect, with a high value for MIC (50 mg ml^−1^) for long chain rhamnolipids (C_14_–C_14_) when tested against *Streptococcus mutans,* to an MIC value as low as 100 µg ml^−1^ with lactonic sophorolipids when tested against *A. naeslundii* or even lower at 40 µg ml^−1^ in the case of *N. mucosa* when treated with the lipopeptide polymyxin. These observed variations are due to structure–activity differences of the selected biosurfactant molecules, which include the synergistic effect of the different congener components within one sample (Das et al. 2014) and the overall product purity. Upon serially diluting the content of the well where no resazurin colour change was observed (MIC value) a substantial reduction in the bacterial count was observed as detailed in Table 3.

**Table 2:** Determination of the MIC and MBC values of different types of biosurfactants against strains listed. All values are expressed in mg ml^−1^

Bacteria	Rh JBR325		Rh C_14_–C_14_		LSL		ASL		Polymyxin	
	MIC	MBC	MIC	MBC	MIC	MBC	MIC	MBC	MIC	MBC
*S. mutans*	0.4	0.8	50	>50	0.2	0.4	Nil	Nil	0.5	1.024
*S. oralis*	0.2	0.4	0.615	12.5	0.1	0.2	Nil	Nil	Nil	Nil
*S. sanguis*	0.2	0.4	0.615	12.5	0.2	0.4	Nil	Nil	0.064	0.12
*MRSA*	Nil	Nil	Nil	Nil	0.4	1.56	Nil	Nil	0.064	0.064
*A. naeslundii*	0.05	0.312	0.312	0.615	0.1	0.4	Nil	Nil	0.512	1.024

**Table 3:** Log reduction and inhibition (%) at the MIC-determined in the Resazurin-aided microdilution method where resazaurin colour remained unchanged, the well was inspected visually to confirm lack of growth, where possible

Bacteria	Rhamnolipids JBR325 Log reduction (inhibition %)	C_14_–C_14_ Rhamnolipid Log reduction (inhibition %)	Lactonic Sphorolipids Log reduction (inhibition %)	Acidic Sphorolipids Log reduction (inhibition %)	Polymyxin Log reduction (inhibition %)
*S. mutans*	3.02 ± 0.26 (99.99)	2.59 ± 0.16 (99)	5.78 ± 0.22 (99.99)	Nil	4.47 ± 0.07 (99.99)
*S. oralis*	2.83 ± 0.16 (99.85)	2.96 ± 0.11 (99.98)	3.97 ± 0.09 (99.99)	Nil	4.76 ± 0.21 (99.99)
*S. sanguis*	4.05 ± 0.37 (99.99)	2.87 ± 0.22 (99.86)	3.67 ± 0.01 (99.99)	Nil	1.00 ± 0.10 (90.0)
MRSA	Nil	Nil	6.57 ± 0.10 (99.99)	Nil	7.02 ± 0.13 (99.99)
*A. naeslundii*	4.06 ± 0.07 (99.99)	4.71 ± 0.36 (99.99)	5.01 ± 0.34 (99.99)	Nil	4.37 ± 0.005 (99.99)
*N. mucosa*	4.10 ± 0.18 (99.99)	4.2 ± 0.19 (99.99)	4.71 ± 0.23 (99.99)	Nil	8.25 ± 0.12 (99.99)

Log reduction and standard deviation are calculated for n = 4

Other papers have reported the use of different concentrations of resazurin dye, some of which are significantly higher than what has been reported in this study. When these higher resazurin concentrations were tested (data not shown) with an untreated bacterial control, false negative results were observed, which can be attributed to the lack of ability of the bacteria to metabolise resazurin at such high concentrations (Singh et al. 2014).

## Conclusion

A new, easy to use, method has been developed which allows the MIC value for biosurfactants to be determined with a high level of accuracy and reproducibility. Problems with poor solubility of the compound under test have largely been overcome by the incorporation of resazurin into the test since the test now includes a measure of bacterial activity. The test has been validated using known antimicrobial antibiotics against standard test organisms. This technique has potential for use in the testing of a wide range of other compounds.
